# Leveraging MicroED to determine structures of protein-bound synthetic small molecules and natural products

**DOI:** 10.1063/4.0001046

**Published:** 2025-10-27

**Authors:** Jose A Rodriguez

**Affiliations:** UCLA, Los Angeles, CA, USA

## Abstract

Accurate characterization of small molecule-protein interactions is critical for research and discovery of pharmaceutically relevant molecules. X- ray crystallography facilitates atomic views of such complexes, is amenable to high throughput approaches, and can rapidly inform drug development. However, the size of crystals that can be routinely interrogated by such high throughput X-ray crystallographic methods can be limiting. Alternatively, serial crystallography approaches or micro electron diffraction (MicroED) can be readily applied to nanocrystals and benefit from the advantages afforded by directly soaking small crystals with ligand solutions. I will discuss the application of MicroED, combined with native mass spectrometry (ED-MS), as a potentially useful approach to rapidly and accurately determining structures of protein-bound small molecules. Specifically, the natural product, E64 and its biosynthetic variants, bound to their target protease, papain. I will show that E64-papain complex structures can even be determined from microcrystals soaked with ligand mixtures and crude biosynthetic reactions, demonstrating the broad utility of ED-MS for research, drug discovery, and medicinal chemistry.

